# Emerging Methodologies in Pediatric Palliative Care Research: Six Case Studies

**DOI:** 10.3390/children5030032

**Published:** 2018-02-26

**Authors:** Katherine E. Nelson, James A. Feinstein, Cynthia A. Gerhardt, Abby R. Rosenberg, Kimberley Widger, Jennifer A. Faerber, Chris Feudtner

**Affiliations:** 1 Pediatric Advanced Care Team, Department of Paediatrics, Hospital for Sick Children, Toronto, ON M5G 1X8, Canada; katherine.nelson@sickkids.ca (K.E.N.); kim.widger@utoronto.ca (K.W.); 2 Institute of Health Policy, Management, and Evaluation, University of Toronto, Toronto, ON M5T 3M6, Canada; 3 Adult and Child Consortium for Health Outcomes Research and Delivery Science, Children’s Hospital Colorado, Aurora, CO 80045, USA; james.feinstein@ucdenver.edu; 4 Division of General Pediatrics, University of Colorado Anschutz Medical Campus, Aurora, CO 80045, USA; 5 Center for Biobehavioral Health, The Research Institute at Nationwide Children’s Hospital, Columbus, OH 43205, USA; cynthia.gerhardt@nationwidechildrens.org; 6 Departments of Pediatrics and Psychology, The Ohio State University, Columbus, OH 43210, USA; 7 Department of Pediatrics, University of Washington School of Medicine; Cancer and Blood Disorders Center, Seattle Children’s Hospital, Seattle, WA 98105, USA; abby.rosenberg@seattlechildrens.org; 8 Treuman Katz Center for Pediatric Bioethics, Seattle Children’s Research Institute, Seattle, WA 98101, USA; 9 Lawrence S. Bloomberg Faculty of Nursing, University of Toronto, Toronto, ON M5T 1P8, Canada; 10 Department of Pediatrics, The Children’s Hospital of Philadelphia, Philadelphia, PA 19104, USA; faerberj@email.chop.edu; 11 Pediatric Advanced Care Team, The Children’s Hospital of Philadelphia, Philadelphia, PA 19104, USA; 12 Department of Pediatrics, The Perelman School of Medicine at the University of Pennsylvania, Philadelphia, PA 19104, USA

**Keywords:** pediatric palliative care, research methods, outcomes

## Abstract

Given the broad focus of pediatric palliative care (PPC) on the physical, emotional, and spiritual needs of children with potentially life-limiting illnesses and their families, PPC research requires creative methodological approaches. This manuscript, written by experienced PPC researchers, describes issues encountered in our own areas of research and the novel methods we have identified to target them. Specifically, we discuss potential approaches to: assessing symptoms among nonverbal children, evaluating medical interventions, identifying and treating problems related to polypharmacy, addressing missing data in longitudinal studies, evaluating longer-term efficacy of PPC interventions, and monitoring for inequities in PPC service delivery.

## 1. Introduction

Pediatric palliative care (PPC) has an expansive mission. In our care of children with potentially life-limiting illnesses, we take into consideration the physical, emotional, spiritual, and relational needs of children and their families [1]. From a clinical perspective, the task of identifying and addressing needs in so many domains is difficult; developing a research agenda that includes all relevant areas of study is equally challenging. There are many important questions that we need to answer as a field. While identifying the question is usually straightforward, designing a high-quality study to answer that question is often tricky. Fortunately, the science of research design is evolving, and novel methodologic approaches can make the barriers to PPC research less daunting. As experienced PPC researchers, we have each faced the gap between defining the question of interest and identifying how best to study it. For this manuscript, we have selected challenges that we have encountered in our own work and identified potential methodologic approaches to address each of them. Our objective is to describe approaches to a broad range of issues encountered in PPC research, but the list is based on our own areas of expertise, and as such, is not comprehensive. We have highlighted other palliative care studies (adult or pediatric) that utilize the proposed methods whenever possible. Specifically, this manuscript describes approaches to: assessing symptoms among nonverbal children, evaluating medical interventions, identifying and treating problems related to polypharmacy, addressing missing data in longitudinal studies, evaluating longer-term efficacy of PPC interventions, and monitoring for inequities in PPC service delivery. The goal of this paper is to motivate novice and experienced researchers by highlighting specific examples of state of the art methods to address some of the current challenges in PPC research. 

## 2. How Can We Assess Pain and Other Symptoms in Nonverbal Children? 

### 2.1 Significance of the Issue 

Symptom burden is a persistent problem among children receiving PPC, necessitating better assessment, communication, and management. Children near end of life experience both physical (such as fatigue, pain, dyspnea, cachexia, and nausea [2,3,4]) and emotional symptoms (for example, sadness, anxiety, and irritability [4,5]). Progressive metabolic conditions may have organic behavioral symptoms that can become increasingly difficult to treat [6]. Further, symptoms often overlap [2,3,5], with fatigue and pain occurring in nearly all children at end of life [2,7,8]. Accurate symptom assessment is the first step toward better management. However, some children are unable to communicate verbally, which complicates this assessment. Inaccurate evaluation of symptoms may result in inadequate treatment and/or unnecessary interventions and polypharmacy. Furthermore, poorly managed symptoms can affect quality of life, satisfaction with care, and parent well-being years later [9,10,11,12,13].

### 2.2 Methodologic Challenges

Methodologic issues have impeded progress in symptom assessment among children receiving PPC. A major barrier is the lack of standardized tools with established psychometric properties. Historically, symptom measures have been created for a specific study or extrapolated from work with adults. Often the format and content are not tailored to the child’s condition or developmental level. Nor have studies consistently reported on other aspects of the symptom (such as type, frequency, severity, or degree of distress [4]). Most studies focus on cancer and rely heavily on symptom tallies from chart reviews or retrospective mother and nurse report. The views of fathers are usually absent, and only one study has included child self-report [14]. While nurses often assume the primary role for symptom assessment and management, nursing and parental reports of children’s symptoms are rarely compared. Additionally, we need to better understand symptom trajectories and identify predictive associations with other quality indicators (such as quality of life/death, satisfaction with care, and parent outcomes).

### 2.3 State of Art or Novel Approaches to Surmount Challenges 

Methodologically rigorous research is essential to lessen symptom burden among children receiving PPC. Measures should: (a) be standardized; (b) include multiple informants, particularly children, mothers, and fathers; (c) encompass diverse health conditions; and (d) be collected prospectively. Obtaining patient-reported outcomes may be difficult for children who are nonverbal due to developmental level, disease progression, or treatment (for example, tracheotomy, pain medication). However, training research staff to assist children using accommodative strategies (such as reading questions aloud, utilizing communication boards/electronic touch pads/adaptive devices) can minimize problems due to fatigue, cognitive deficits, or physical ability. Direct observation and brief ecological momentary assessment through phone call, texting, or short online surveys can also help. If children cannot reliably report, proxy-reports of parents or nurses may be required. A mixed method and multi-informant approach is typically recommended. 

Pediatric palliative care research to characterize symptomatology, identify associations with other outcomes, and evaluate interventions is now underway. The study Charting the Territory includes longitudinal symptom assessment among children with progressive, metabolic, neurological, or chromosomal conditions [15]. The Pediatric Quality of Life and Evaluation of Symptoms Technology (PediQUEST) study has examined the efficacy of providing real-time feedback to providers about electronic patient-reported outcomes on symptom burden and quality of life in children with advanced cancer [14,16]. With continued efforts and attention to methodological rigor, we can achieve meaningful progress in symptom management for children with life-limiting conditions.

## 3. How Can We Assess the Impact of Medical Interventions on Children with Serious Illness? 

### 3.1 Significance of the Issue

One challenge in providing PPC is the limited evidence base for treatments [17]. We often rely on the adult literature about effective interventions and therapies, despite the potential risks associated with “off-label” medication use [18] and challenges in translating adult data for pediatric use [19]. Similarly, the adoption of procedures may outpace the evidence base. For example, a 2013 Cochrane review of gastrostomy tube feeding in children with cerebral palsy did not identify any studies meeting inclusion criteria, concluding that “considerable uncertainty about the effects of gastrostomy tube feeding for children with cerebral palsy remains” [20]. Despite this uncertainty, gastrostomy tubes are common: in two population-based studies of children with neurologic impairment, approximately 10% had gastrostomy tubes [21,22]. 

### 3.2 Methodologic Challenges

While ideally every intervention would be subjected to a large, high quality pediatric randomized controlled trial, ethical challenges, identification of adequately sized samples, and funding issues often interfere [23,24]. Also, children with complex medical conditions may not meet inclusion criteria for many trials [25]. A study of clinical trials in adolescent depression found that nearly 70% excluded children with “any current significant physical condition” [26]. Recruiting families for PPC-specific trials can also be difficult [27]. For these reasons, health administrative data has emerged as an appealing alternative for bolstering the evidence base for treatments in pediatrics [28]. However, this alternative is not without its challenges. While these data sources are population-based—with real-world outcomes and large samples, even for relatively rare diseases—they also pose challenges. For children with complex disease or multiple comorbidities, the granular clinical details necessary to identify comparable intervention and control groups are often lacking. Therefore, in PPC studies using health administrative data, population heterogeneity is often a significant issue. 

### 3.3 State of Art or Novel Approaches to Surmount Challenges

Among several options for managing heterogeneous populations in health administrative data are two fairly new methodologies that may be helpful in PPC research design. First, since identifying an appropriate control group is often impossible, one option is to use each individual as his or her own control. In an exposure-crossover design, the rate of a recurrent outcome is compared before and after an exposure [29]. Because every individual has the same profile of fixed characteristics before and after the exposure, the design itself controls all patient-level factors that are stable over time. Self-matching is useful for estimating population-wide effects but has limited ability to evaluate the influence of clinical characteristics, which can vary over time. For these situations, longitudinal models that allow the values of clinical covariates to change over time are required. Second, in a heterogeneous population the question is often about differential effects across subgroups. In latent class analysis and other mixture models, the statistical model can group individuals into classes based on clusters of traits that predict outcomes [30]. In traditional regression models, the independent effect of each trait on the outcome is estimated, whereas latent class analysis identifies patterns in the combined effects of multiple traits on the outcome, allowing a richer description of subgroups within a heterogeneous population [31], as exemplified in two recent PPC studies [32,33]. Latent class analysis can identify potential subgroups of patients, sharing similar observed characteristics, who might experience different effects from a given treatment. This approach is similar to traditional subgroup analysis but surmounts some of the methodological challenges of sub-group analyses, including low statistical power, falling prey to type I errors, and failing to identify higher-order treatment effect interactions [31]. Thus, methodologies like the exposure-crossover design and latent class analysis can be helpful to manage population heterogeneity in studies evaluating the effectiveness of interventions among children receiving PPC.

## 4. How Do We Monitor and Manage the Problems Arising from Polypharmacy? 

### 4.1 Significance of the Issue

The goal of pharmacotherapy in children receiving PPC is to alleviate existing symptoms while minimizing side effects. Children receiving PPC may have increasing polypharmacy as their physical symptoms progress [34]. Children with polypharmacy may take five or more medications simultaneously, and in PPC these regimens frequently include multiple high-risk medications (such as opioids, psychotherapeutics, and anticonvulsants) [34,35,36,37]. Problems from polypharmacy may include increased risk for side effects, adverse drug events, drug-drug interactions, and drug-disease interactions [36,37,38,39,40]. Evidence to guide pharmacotherapy for children receiving PPC is limited for single medications and almost non-existent for complicated regimens.

### 4.2 Methodologic Challenges

Methodologic challenges complicate detection and management of problems from polypharmacy. First, few population-level data sources contain information about exposures (medications) and outcomes (adverse drug events). Infrequent adverse drug events represent “needles in the haystack,” and large comprehensive data sources are required to complete pharmacoepidemiologic studies in populations with rare conditions. Second, identifying outcomes is challenging in both population and clinical studies. Few outcome measures—either case definitions of adverse drug events or patient-reported outcome measures—are available in pediatrics, let alone for children receiving PPC. Third, children receiving PPC may experience multiple treatment- and condition-related symptoms, and this overlap complicates the detection of adverse drug events. Finally, even when evidence exists to support management strategies, translating generalized recommendations to unique and complex PPC patients is difficult [41,42].

### 4.3 State of Art or Novel Approaches to Surmount Challenges

Fortunately, recognition of the risks of polypharmacy has driven the development of new approaches to meet these challenges. Enhanced data sources now permit population-level pharmacoepidemiologic studies, including in ambulatory settings [35,36,37]. National data sources provide large enough sample sizes to analyze patterns of medication exposure in patients with rare diseases [43], and the increasing availability of integrated clinical data may allow for better detection of adverse drug events. Furthermore, big data algorithms allow for the assessment of exposures—including exposures to multiple drug combinations—at increasingly smaller intervals (such as on a daily or even hourly basis) [35]. Improved temporal resolution will increase our ability to understand the sequence of events leading to problematic polypharmacy, allowing us to target interventions toward periods of increased risk.

Limited availability of objective patient data complicates the identification of adverse drug events in clinical studies. The Food and Drug Administration’s Best Pharmaceuticals for Children Act has prioritized the development of outcome measures of drug safety and efficacy in children with intellectual and developmental disabilities [38,44]. Patient- and proxy-reported symptom assessments developed for PPC patients [14,16] are now being studied for their specific utility in assessing symptom variation before and after medication changes. Patient-Reported Outcomes Measurement Information System (PROMIS) is also expanding the availability of pediatric-specific patient-reported outcome measures, even for those children with neurologic impairment [45,46]. Utilization of these measures may identify changing symptomatology, which can guide therapeutic decisions and enhance medication safety [14,47]. Ultimately, evolving multi-center PPC research networks will enable more rigorous evaluations of medication management strategies specific to PPC patients [41,48].

## 5. How Can We Address Problems Arising from Missing Data in Longitudinal Studies? 

### 5.1 Significance of the Issue 

Longitudinal studies of PPC patients are particularly prone to missing data because PPC patients have a high level of morbidity and mortality, and are thus often unable to complete assessments, especially as their illness advances or their symptoms worsen. The subsequent presence of missing data in these studies erodes precision and threatens the validity of results. Said differently, the loss of precision results in decreased statistical power to detect meaningful differences in effects, and reasons why the data are missing can introduce potential biases in the estimation of effects [49]. If we ignore the presence of missing data in a PPC study, and children who are sicker are more likely to have missing data, inferences made based on data from the remaining less-ill or less-symptomatic participants can be misleading, failing to reflect what happened to all the participants, and may over- or under-estimate associations or treatment effects.

### 5.2 Methodologic Challenges

Analytic approaches for handling missing data are all based on three different possible assumptions about the mechanisms that can result in missing data: missing completely at random (MCAR), missing at random (MAR), and missing not at random (MNAR). The analytic approach of last observation carried forward is easy to implement but should be avoided even when missing data are MCAR [50]. The analytic approaches of complete-case analysis and unweighted generalized estimating equations make a strong assumption that the data are MCAR (that is, missing data is independent of both observed and unobserved data [51]), an assumption that is unrealistic in a PPC study of patients with high-levels of symptom burden, psychosocial stress, and death. MCAR methods might be sufficiently valid with a small amount of missing data (<5%) but otherwise can lead to serious bias and loss of precision [52]. If the data is assumed to be MAR (that is, missingness depends only on observed data), there are several practical approaches for handling missing data [53], including multiple imputation (MI), weighted procedures that adjust for drop out, and maximum likelihood model-based approaches, such as mixed-effects models. Of note, for these approaches to be accurate, other assumptions (such as no model misspecification) need to be met, and MI requires large sample sizes and strong covariates [49]. Methods exist to test whether the data are MCAR or MAR using data from the study but, importantly, no test exists to rule out the possibility that the data are actually MNAR (that is, missingness is related to unobserved characteristics, such as an unexpected progression of disease or worsening of symptoms, even after accounting for observed data). Importantly, if the missing data is believed to be MNAR—which is most often likely—the missing data mechanism should not be ignored [49].

### 5.3 State of Art or Novel Approaches to Surmount Challenges 

Ideally, the impact and potential bias of missing data is reduced by how the study is designed [52]. This can be done by enabling multiple ways to collect not only the primary data regarding participants, but also auxiliary data. By this phrase “auxiliary data,” we mean data collected expressly to address problems that will arise from the expected degree of missingness of primary data elements. Auxiliary data need to be associated with the outcome and should be easier or more feasible to collect than the outcome data. Some approaches to gathering auxiliary data include administration of a short assessment containing only three to five items that are presumed to be highly correlated with the primary outcomes; obtaining assessments from other informants; and measuring variables that might be surrogate outcomes (which is to say, an outcome that is not the most valued main outcome, such as reduction in the level of a symptom, but instead a proxy outcome or a process measure that is strongly associated with the valued main outcome, such as a biomarker or the receipt of an effective symptom ameliorating medication) [49,52].

If the missing data might be MNAR, the methods described above under the MAR assumptions can be used for the primary analysis of incomplete longitudinal data, as these models are robust to departures from MAR [50], but additional analyses using MNAR models should be performed to examine the extent to which inferences from the primary outcome model vary across the different scenarios for missing data. There are three main approaches to performing these sensitivity analyses [54]: selection models [51], pattern-mixture models [55,56], and shared random effects models. These methods pose their own challenges [57], but we believe that the combination of MAR models and sensitivity analyses using MNAR models is the best way to advance our knowledge and understanding in the face of missing data in PPC studies.

## 6. How Can We Assess Longer-Term Outcomes of Interventions? 

### 6.1 Significance of the Issue 

Studies comparing children with and without PPC involvement suggest that PPC results in improved symptom management, fewer invasive procedures, and more comprehensive anticipatory end-of-life guidance [58,59,60]. The few published randomized controlled trials (RCTs) evaluating PPC interventions also demonstrate improved symptom burden and quality of life, clearer adolescent goals of care, and improved parental mood [16,61,62,63]. All studies included outcome measurements at specific time-points after the intervention. Among the RCTs, the longest follow-up was 9 months, and investigators observed substantial participant-attrition over time [16,48]. Although proximal efficacy is important, the lack of long-term observation represents a crucial gap in knowledge [64]. Trajectories of serious pediatric illness are long-lasting and characterized by fluctuating and/or chronic symptoms, varying acuity and needs, and diverse psychosocial sequelae [17,34,65,66,67]. Furthermore, parents and healthy siblings are also at risk for delayed consequences (such as poor mental health and risky health behaviors) [68,69,70,71]. Improving these outcomes demands that we also evaluate the durability of PPC interventions among patients and their families. 

### 6.2 Methodologic Challenges

Demonstrating long-term benefit will not be easy. First, adult studies suggest palliative care interventions may have short-lived effects; a recent meta-analysis of RCTs showed improved quality of life and symptom burden at 3-months, but not at 6-months [72]. Second, measuring quality of life among children with serious illness is challenging, particularly since validated instruments are limited [73]. Third, outcomes beyond quality of life are important; we also wonder about patients’ functional outcomes (for example, return to school), parent and sibling outcomes (such as caregiver burden), sibling outcomes (such as adjustment), and experience-specific sequelae (such as bereavement outcomes). Fourth, relevant follow-up times depend on patient trajectories, which vary across individuals and diagnoses. Additionally, the possibility of external factors modifying outcomes increases with longer follow-up times. Finally, if findings suggest minimal long-term efficacy, the appropriate response is unclear: are improved short-term outcomes sufficient to warrant implementation, or do we delay implementation until we develop boosters to extend efficacy?

### 6.3 State of Art or Novel Approaches to Surmount Challenges 

Addressing these challenges requires a multi-faceted approach. We should determine what is important to stakeholders, when it is important, and how we can best measure it, which may require the development of new instruments. Due to variability in patient and family- experiences, studies must be longitudinal and include repeated measures. Rather than describing scores at isolated time-points, novel approaches might include “areas under the curve” or comparisons of trajectories between patients who do or do not receive interventions. Investigators should consider interdisciplinary and community partnerships to facilitate longer-term follow-up (for example, partnering with teachers to assess bereaved siblings [70]). Lastly, we must leverage cooperative research groups to enroll sufficient numbers of children and families. Serious illness in children endures. It is time to determine if and how the impact of PPC interventions endures too. 

## 7. How Do We Monitor for Inequities in the Provision of PPC? 

### 7.1 Significance of the Issue 

The World Health Organization promotes a “vision of a future in which all people have access to health services that are provided in a way that are coordinated around their needs, respects their preferences, and are safe, effective, timely, affordable, and of acceptable quality” [74]. Unfortunately, in PPC we face many unanswered questions about inequities in PPC provision. International research indicates that not all children who might benefit from PPC receive it. Reported PPC referral rates for children who died after any life-threatening condition vary widely across countries and institutions, with reported rates ranging from 8% to 39% [75,76,77,78,79,80]. Among children with cancer, rates tend to be higher, with a 63% referral rate reported in one Canadian study [80]. The evaluation of characteristics between children who do and do not receive PPC identifies concerning inequities. Referral rates are lower among children living in rural areas or low-income neighborhoods [80] as well as for African-American children and those without private insurance [76]. Studies show that in child health overall [81], as well as in PPC [82], barriers to accessing appropriate services are multilevel, requiring intervention at the level of the child and family, healthcare provider, health system, and broader society [81,82]. 

### 7.2 Methodologic Challenges

Inequities in receipt of PPC have been identified primarily through the use of population-based health administrative data [76]. However, administrative data are not available everywhere, and they do not show the origin of the disparity (for example, healthcare providers may preferentially offer PPC or certain families may be more likely to decline PPC involvement). Misclassification is also possible: families might receive excellent PPC through community providers, and this care may not be identified in administrative data. Additionally, for families who decline PPC, we know little about individual-level (such as lack of knowledge) or societal-level (such as general distrust of the healthcare system) factors that may affect their decision-making [81]. 

### 7.3 State of Art or Novel Approaches to Surmount Challenges 

Despite the limitations of currently available health administrative data, continued use of these data will help assess the effect of interventions to address disparities. Locally, or where administrative data are not available, comparing demographic characteristics (such as ethnicity, race, language spoken, income, geographic area) among referred children to those of the population served by the institution may highlight potential inequities [83]. Alternatively, as part of morbidity and mortality rounds for children who die within an institution, differences in demographic characteristics between children who did and did not receive PPC could be examined. Further evaluation of disparities in PPC provision may also be facilitated by development, implementation, and validation of “trigger” systems through electronic medical records to identify children who have been diagnosed with a life-threatening condition based on published International Classification of Diseases, Tenth Revision (ICD-10) codes [39,84,85,86,87], or other disease- or family-related factors [88]. Such systems may help overcome referral biases among health professionals. They also offer the opportunity to explore decision-making when the system is triggered but a referral does not happen. Qualitative research to explore the views of health providers and families when referrals do not happen would allow for the identification of contributing factors, which could further help with the monitoring and guiding of efforts to reduce inequities. 

## 8. Conclusions

As PPC providers, we pride ourselves in finding creative solutions to seemingly intractable clinical challenges. To address the seemingly unanswerable research questions that are ubiquitous in PPC, we should apply the same “think outside the box” approach to our methodologies. Pushing the boundaries of research design will help us provide the best possible care to children with potentially life-limiting illnesses and their families. 
